# Differential DNA methylation associated with multiple sclerosis and disease modifying treatments in an underrepresented minority population

**DOI:** 10.3389/fgene.2022.1058817

**Published:** 2023-01-04

**Authors:** Jeremy M. Bingen, Lindsay V. Clark, Mark R. Band, Ilyas Munzir, Michael D. Carrithers

**Affiliations:** ^1^ Neurology, University of Illinois College of Medicine, Chicago, IL, United States; ^2^ Physiology and Biophysics, University of Illinois College of Medicine, Chicago, IL, United States; ^3^ High Performance Biological Computing, and Roy J Carver Biotechnology Center, University of Illinois, Champaign, IL, United States; ^4^ Neurology, Jesse Brown Veterans Administration Hospital, Chicago, IL, United States

**Keywords:** epigenetics, biomarker, black, african American, hispanic, latino, dimethyl fumarate

## Abstract

Black and Hispanic American patients frequently develop earlier onset of multiple sclerosis (MS) and a more severe disease course that can be resistant to disease modifying treatments. The objectives were to identify differential methylation of genomic DNA (gDNA) associated with disease susceptibility and treatment responses in a cohort of MS patients from underrepresented minority populations. Patients with MS and controls with non-inflammatory neurologic conditions were consented and enrolled under an IRB-approved protocol. Approximately 64% of donors identified as Black or African American and 30% as White, Hispanic-Latino. Infinium MethylationEPIC bead arrays were utilized to measure epigenome-wide gDNA methylation of whole blood. Data were analyzed in the presence and absence of adjustments for unknown covariates in the dataset, some of which corresponded to disease modifying treatments. Global patterns of differential methylation associated with MS were strongest for those probes that showed relative demethylation of loci with lower M values. Pathway analysis revealed unexpected associations with shigellosis and amoebiasis. Enrichment analysis revealed an over-representation of probes in enhancer regions and an under-representation in promoters. In the presence of adjustments for covariates that included disease modifying treatments, analysis revealed 10 differentially methylated regions (DMR’s) with an FDR <1E-77. Five of these genes (ARID5B, BAZ2B, RABGAP1, SFRP2, WBP1L) are associated with cancer risk and cellular differentiation and have not been previously identified in MS studies. Hierarchical cluster and multi-dimensional scaling analysis of differential DNA methylation at 147 loci within those DMR’s was sufficient to differentiate MS donors from controls. In the absence of corrections for disease modifying treatments, differential methylation in patients treated with dimethyl fumarate was associated with immune regulatory pathways that regulate cytokine and chemokine signaling, axon guidance, and adherens junctions. These results demonstrate possible associations of gastrointestinal pathogens and regulation of cellular differentiation with MS susceptibility in our patient cohort. This work further suggests that analyses can be performed in the presence and absence of corrections for immune therapies. Because of their high representation in our patient cohort, these results may be of specific relevance in the regulation of disease susceptibility and treatment responses in Black and Hispanic Americans.

## Introduction

Multiple sclerosis (MS) is a major cause of non-traumatic neurologic disability in young adults. The prevalence of MS is increasing worldwide and is more common in underrepresented minority groups than previously thought (Weinstock-Guttman et al., 2003; Cree et al., 2004; Chinea et al., 2012; Caldito et al., 2018; Wallin et al., 2019). Although non-Hispanic Whites still have the highest prevalence rate for MS in the US, the demographics of newly diagnosed MS are also changing. One study of patients in the US demonstrated that Black American women had the highest incidence of MS and that Black men had a similar incidence as compared to White, non-Hispanic men (Langer-Gould et al., 2013). Analysis of the Gulf War military-veteran cohort also demonstrated a higher incidence of MS in Black Americans than other demographic groups (Wallin et al., 2012).

In addition, multiple studies have demonstrated increased disease severity and risk of long-term disability in Black American patients (Cree et al., 2004; Caldito et al., 2018; Wallin et al., 2018). Although studies in the modern era suggest that disease modifying treatments and improved diagnosis are associated with decreased long-term severity of MS (Sorensen et al., 2020), these observations may not be relevant to minority populations. These disparities in clinical outcomes and treatment responses may reflect social and environmental determinants of health as has been shown for other chronic diseases.

These determinants of health may impact the epigenome. One example is the regulation of DNA methylation, which is a dynamic process throughout the lifetime of an individual (Li and Zhang, 2014). The rationale for the study of epigenetic mechanisms in MS is that environmental factors such as stress, diet, and environmental exposures are all known modulators of DNA methylation. Some of these epigenetic mechanisms are associated with chronic inflammatory states (Celarain and Tomas-Roig, 2020). Most prior studies of global DNA methylation in MS have focused on individuals of Northern European ancestry. As in genome wide association studies (GWAS), the strongest association between MS and differential DNA methylation occurs at the HLA-DRB locus (Kular et al., 2018).

The approach in this study was to evaluate differential DNA methylation in a cohort of patients that are predominantly from underrepresented minority groups. This cohort is from our clinical practice at the University of Illinois, Chicago where approximately 55% of patients identify as Black or African American and 25% as Hispanic or Latino. The primary goal of this work was to identify epigenetic markers and related cellular signaling mechanisms that are associated with disease susceptibility in our patient population. In addition, challenges for the characterization of epigenetic biomarkers in a real-world setting is that most patients are on disease modifying treatments which may also regulate DNA methylation. An additional goal was to demonstrate the feasibility of identifying epigenetic biomarkers of disease and treatment in parallel analyses.

## Results

### Clinical phenotype variance in the MS cohort

MS patients (n = 29) and controls (n = 18) were recruited from our clinical practice at the University of Illinois, Chicago. A summary of demographic data for each group is shown in Table 1, and more detailed demographic and clinical data for each MS patient are shown in Supplmentary Table S1. Phenotypic variance of this patient cohort is shown in Supplmentary Figure S1 based on Functional Systems Scores. More extensive clinical phenotyping using network analysis has been performed on a larger number of patients from the same cohort (Howlett-Prieto et al., 2022).

**TABLE 1:** Donor demographics for methylomic studies.

Group	N =	Age±SD	%Female (%)	%Black or african american (%)	%White, hispanic american (%)	%White, non-hispanic (%)
MS	29	43 ± 11	69	73	24	3
Control	18	43 ± 14	67	50	39	11

### Differential DNA methylation between MS and controls at specific probe sites

**FIGURE 1:**
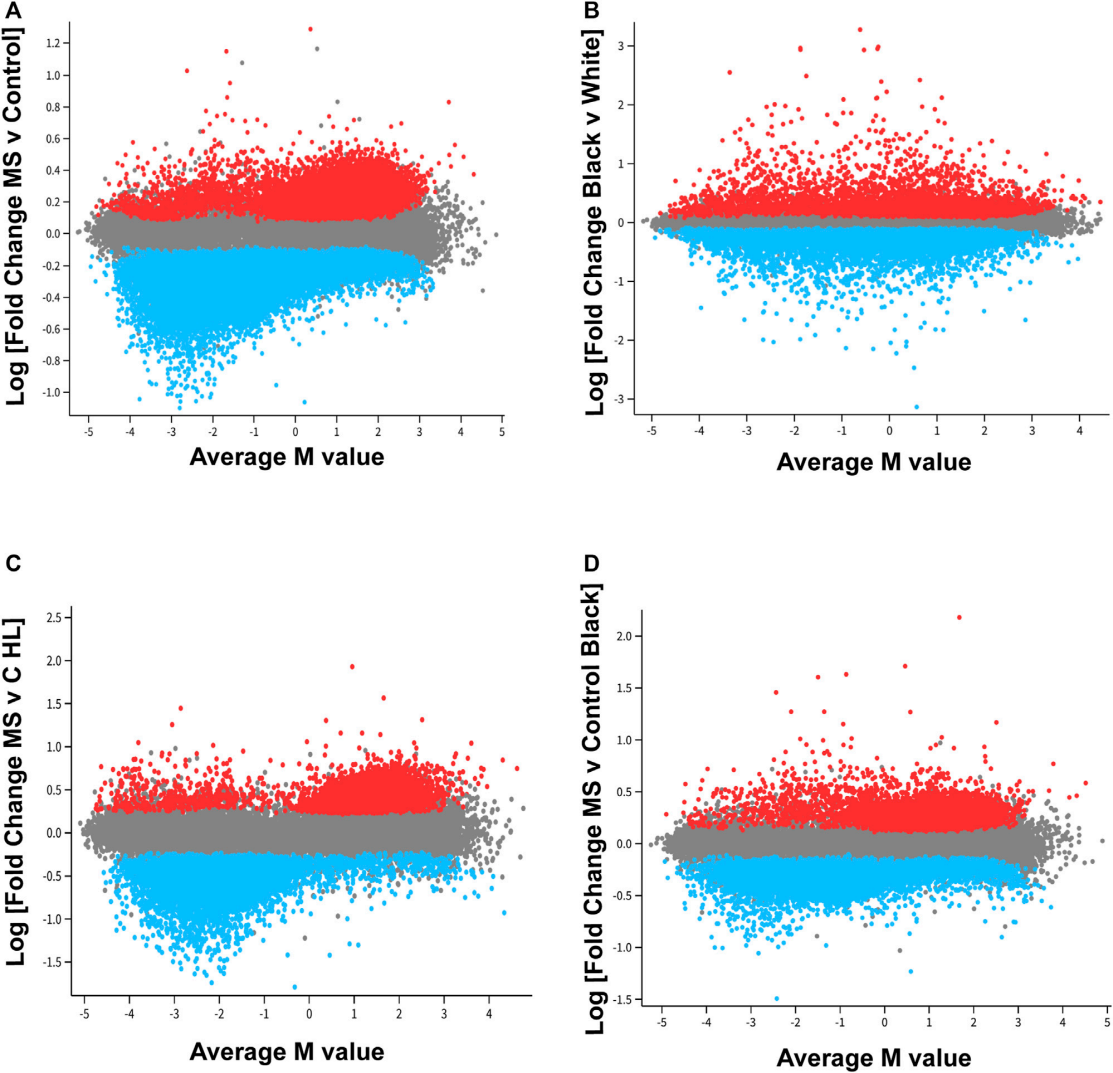
Distinct pattern of differential methylation of genomic DNA associated with MS. In the mean difference (MD) plots, average M-values across all donors is plotted on the x-axis. A negative number on the x-axis indicates decreased methylation for that locus [differentially methylated probe, DMP; CpG region), whereas as a positive number designates increased methylation as compared to other loci. Log fold change is plotted on the y-axis. **(A)**] For the MS *versus* control comparison, 52,295 DMP’s were included in this analysis (FDR<0.01). An additional 20,000 probes were randomly selected for inclusion in the plot (gray). Red points designate probes that showed a relative increase in methylation MS as compared to controls, and blue points represent probes that demonstrated a relative decrease in methylation in the MS group. Two trends were observed. For those probes with the greatest fold change differences [Log (FC) > 0.7 or < −0.7, fold change of greater 5×, FDR<0.01], these differences primarily reflected decreased methylation of those DMP’s with lower average M-values (M value <0; blue, left lower quadrant). If a less stringent cut off was used for fold change, the results suggested a tendency for significant DMP’s that had an average methylation score over 50% (Average M value >0) to be hypermethylated in MS patients, and significant probes that had an average methylation below 50% to be hypomethylated in MS patients. **(B)** These trends were not observed in a comparison of all Black donors *versus* all White donors. The racial differences showed a more normalized distribution of relative increases or decrease in probe methylation as compared to the MS *versus* control analysis. **(C,D)**. MD plots were also performed in racial and ethnic subgroups. The trend observed in the MS *versus* control comparison was most pronounced in the Hispanic-Latino subgroup.

The next goal was to analyze patterns of differential DNA methylation between control and MS donors. As described in Methods, probes were filtered (n = 788,804) and adjusted for gender, age, and unknown covariates, some of which corresponded to disease modifying treatment. Adjusted M-values were used to generate Mean Difference (MD) plots (Figure 1) (Su et al., 2017).

Analysis revealed distinct patterns of global differential methylation (Figure 1A, blue represents loci that are demethylated in MS *versus* controls and red increased methylation). For those DMP’s (differentially methylated probes for a specific CpG region) with the greatest fold change differences [Log (FC) > 0.7 or < -0.7, fold change of greater 5x, FDR<0.01], these differences primarily reflected decreased methylation of those DMP’s with lower average M-values (M value <0; blue, left lower quadrant). There were 174 DMP’s that met these criteria in the left lower quadrant, 10 in the upper left, 1 in the lower right, and 4 in the upper right (Supplementary Table S2). These results suggested that those probes with the greatest differences between MS and controls were associated with demethylation of loci that have relatively low levels of methylation across all donors**.**

In contrast, global differential methylation patterns showed a more normalized distribution of relative increases or decreases of probe methylation in a comparison of all Black donors *versus* all White (Hispanic and non-Hispanic) individuals (Figure 1B). Global patterns of methylation were also analyzed in demographic subgroups (Figures 1C,D). These data suggested that the global pattern observed for MS *versus* Control (Figure 1A) occurs in both MS comparator groups but is most marked in the Hispanic-Latino group (Figure 1C).

### Comparison of differentially methylated probes between racial and ethnic groups

The top 10,000 DMP’s (Supplementary Tables S3–S5) for each comparator group (MS *versus* Control for all patients, MS *versus* Control Black American only, and MS *versus* Control Hispanic-Latino only) were analyzed to assess common and distinct probe sets (Supplementary Figure S2). There were 20,518 probes that were present in at least one of the comparator groups. We further identified 4395 probes unique for the Black American group and 6,025 probes for the Hispanic-Latino group (Supplementary Tables S6,S7). These data were not adjusted for unknown covariates.

KEGG pathway analysis was performed on the Top 10,000 DMP’s for each comparator group (Supplementary Tables S8–S10). In the comparison between all patients, there was a possible association with sphingolipid and T cell signaling pathways (FDR = 0.01). In the Hispanic-Latino group, there was a possible association with apelin signaling (FDR = 0.07) which was not observed in the other comparator groups. Pathway analysis of the probes unique for the subgroups (Supplementary Figure S1) did not yield any statistically significant associations (FDR>0.18 for the Hispanic-Latino group and FDR>0.68 for the Black American group).

More robust results were obtained for pathway analysis following the removal of CpG regions associated with methylation quantitative trait loci (mQTL). Although known SNP regions were filtered prior to analysis, many probes remain in the data set that are associated with genetic variation at CpG loci (Min et al., 2021). For this reason, we performed analysis on subsets of probes that are associated mQTL and those that are not. The MeQTL Epic database (https://epicmeqtl.kcl.ac.uk) was utilized to identify mQTL associated with probes for the Illumina Infinium MethylationEPIC array (Villicana and Bell, 2021). In the comparison between all patients, 6,577 loci were identified that were not associated with mQTL (Supplementary Table S11). KEGG pathway analysis revealed 20 pathways potentially associated with differential methylation in MS (Table 2; *p* < 0.005, FDR<0.10). These pathways included those related to immune function such as hematopoietic cell lineage and chemokine signaling and unexpected associations with bacterial invasion of epithelia, amoebiasis, and shigellosis. Notably, no statistically significant associations were found for viral infections such as Epstein Barr (*p* = 0.64, FDR = 0.90). Significant associations were not observed for loci associated with mQTL or in the demographic subgroup analyses (FDR>0.10).

**TABLE 2:** KEGG pathway analysis of differential methylation in MS at loci not associated with mQTL.

KEGG pathway	Description	N (loci)	DE	P.DE	FDR
path:hsa04640	Hematopoietic cell lineage	91	29	2.91E-05	0.010
path:hsa05100	Bacterial invasion of epithelial cells	76	32	0.0001	0.020
path:hsa04611	Platelet activation	123	44	0.0003	0.021
path:hsa04062	Chemokine signaling pathway	189	55	0.0003	0.021
path:hsa04071	Sphingolipid signaling pathway	118	42	0.0003	0.021
path:hsa05418	Fluid shear stress and atherosclerosis	137	41	0.0003	0.021
path:hsa04973	Carbohydrate digestion and absorption	45	18	0.0007	0.035
path:hsa05146	Amoebiasis	98	33	0.0009	0.035
path:hsa04725	Cholinergic synapse	112	42	0.0009	0.035
path:hsa05131	Shigellosis	238	64	0.0014	0.044
path:hsa04912	GnRH signaling pathway	91	33	0.0014	0.044
path:hsa04750	Inflammatory mediator regulation of TRP channels	97	36	0.0015	0.044
path:hsa04660	T cell receptor signaling pathway	99	34	0.0021	0.056
path:hsa04014	Ras signaling pathway	228	68	0.0024	0.059
path:hsa04072	Phospholipase D signaling pathway	144	50	0.0026	0.060
path:hsa04668	TNF signaling pathway	109	32	0.0029	0.065
path:hsa05200	Pathways in cancer	515	132	0.0034	0.067
path:hsa05144	Malaria	49	15	0.0034	0.067
path:hsa04722	Neurotrophin signaling pathway	114	38	0.0041	0.076
path:hsa05221	Acute myeloid leukemia	64	24	0.0043	0.076

DE: discrete elements (genes); FDR: false detection rate.

### Comparison of differentially methylated probes with genome wide association studies

The International MS Genetics Consortium (IMSGC) reported a detailed analysis of currently available GWAS data identified a list of 551 non-MHC genes considered to be of high priority that are associated with peripheral immune function and microglia (International, 2019). We compared this gene list with our list of top DMP’s (n = 20,518). 43 SNP regions from the prioritized gene list were present in genes that also contained DMP’s at other loci (Supplementary Table S13).

### Analysis of gene regulatory regions reveals over-representation of enhancer regions in MS associated DMP’s

Enrichment analysis was performed to determine if there was an over-representation of enhancer or promoter regions among those DMP’s that were associated with MS. For these analyses, the top 10,000 statistically significant DMP’s (Supplementary Tables S3–S5; n = 20,518) in the 3 comparator groups (MS *versus* Control for All, Black and Hispanic American subgroups) were compared to the proportion of gene regulatory elements in the full data set (n = 788,804). The following databases and regions were analyzed: FANTOM5 (functional annotations of the mammalian genome, version 5) enhancers (Andersson et al., 2014), ENCODE (encyclopedia of DNA elements) annotations for promoter and enhancer regions (Gerstein et al., 2012), TSS200 (transcriptional start site within 200 bp), and TSS1500 (transcriptional start site within 1,500 bp.

These analyses demonstrated over-representation of enhancer regions and reduced frequency of promoter regions in the MS datasets (Table 3). The most striking findings were for over-representation of FANTOM5 enhancer regions (odds ratio 3.90, p < 1e-15, Fisher’s Exact Test for Count Data) and under-representation of ENCODE promoter regions (odds ratio 0.26, p < 1e-15) in the MS *versus* Control (all donors) comparator group. Similar results were observed in the demographic subgroups.

**TABLE 3:** Cell type composition analysis.

Condition	CD8	CD4	NK	B cell	Monocyte	Neutrophil
Control	0.12 ± 0.04	0.13 + 0.03	0.06 + 0.02	0.07 ± 0.04	0.09 ± 0.02	0.56 ± 0.08
Multiple Sclerosis	0.09 ± 0.04	0.10 ± 0.06	0.05 ± 0.02	0.06 ± 0.04	0.10 + 0.02	0.64 ± 0.11

Values are Mean ± standard deviation.

### Cell composition analysis

**FIGURE 2:**
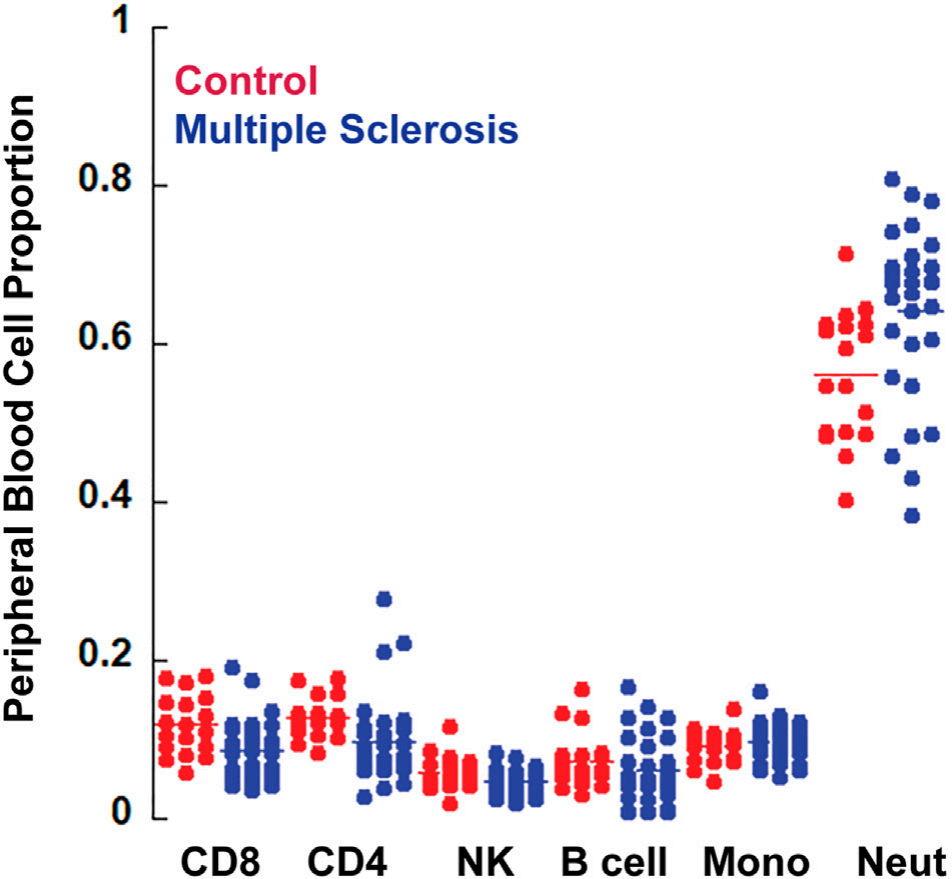
Cell composition analysis. The CellCounts2 function in the FlowSorted.Blood.EPIC package in R software was used to estimate differential peripheral blood cell counts. No statistically significant differences were observed between MS and controls.

Because whole blood methylomics were utilized in this study, some differences may reflect differences in subsets of peripheral blood cells. For that reason, we also performed cell type composition analysis. Methylation data from flow sorted whole blood was used to estimate cell type composition for each sample (Salas et al., 2018). Although there was a trend toward a modest increase in neutrophils and a decrease in CD8 T lymphocytes in the MS group, these differences were not statistically significant (Table 4, Figure 2). Neutrophils were the predominant subtype in both MS and controls.

**TABLE 4:** Enrichment analysis for DNA regulatory regions.

Group	DNA region	DMP proportion		Odds ratio*	*p*-value*
		Top DMP’s in MS n = 10,000	All DMP’s n = 788,804		
MS *versus* Control (All)	Enhancer FANTOM5	0.1295	0.0332	3.90	<1e-15
	Enhancer	0.2101	0.1535	1.47	<1e-15
	ENCODE/X450K				
	TSS200	0.0216	0.0433	0.49	<1e-15
	TSS1500	0.0393	0.0631	0.61	<1e-15
	Promoter associated	0.0325	0.1298	0.26	<1e-15
	ENCODE				
MS *versus* Control (Black or African American)	Enhancer FANTOM5	0.0915	0.0332	2.94	<1e-15
	Enhancer	0.2000	0.1535	1.38	4.33e-13
	ENCODE/X450K				
	TSS200	0.0249	0.0433	0.56	<1e-15
	TSS1500	0.0461	0.0631	0.72	<1e-15
	Promoter associated	0.0362	0.1298	0.25	<1e-15
	ENCODE				
MS *versus* Control (Hispanic or Latino)	Enhancer FANTOM5	0.0766	0.0332	2.42	<1e-15
	Enhancer	0.2053	0.1535	1.42	<1e-15
	ENCODE/X450K				
	TSS200	0.0388	0.0433	0.89	0.026
	TSS1500	0.0510	0.0631	0.80	3.78e-07
	Promoter associated	0.0442	0.1298	0.31	<1e-15
	ENCODE				

* Fisher’s Exact Test for Count Data.

Abbreviations: TSS, transcriptional start site; DMP, differentially methylated probe; FANTOM5, functional annotations of the mammalian genome, version 5; ENCODE, encyclopedia of DNA, elements.

### Differential methylation in HLA-DRB1 region is not associated with MS in our patient population

In addition to these comparisons, we performed analysis of 14 CpG loci within the HLA-DRB1 region which had previously been shown to be differentially methylated in MS patients recruited in Scandinavia and Germany (Kular et al., 2018). Unfiltered and unadjusted M values were utilized for this analysis because ten of the CpGs had to be retrieved from the dataset before filtering. Six of them were within known SNPs, three were known to be cross-reactive, and two (including one of the cross-reactive probes) were not detected in all samples. Out of the 19 CpGs in the region identified by Kular et al., 2018, two were not analyzed here due to being absent from the Infinium MethylationEPIC bead array chip, and three are not shown here because they did not show significant methylation differences.

**FIGURE 3:**
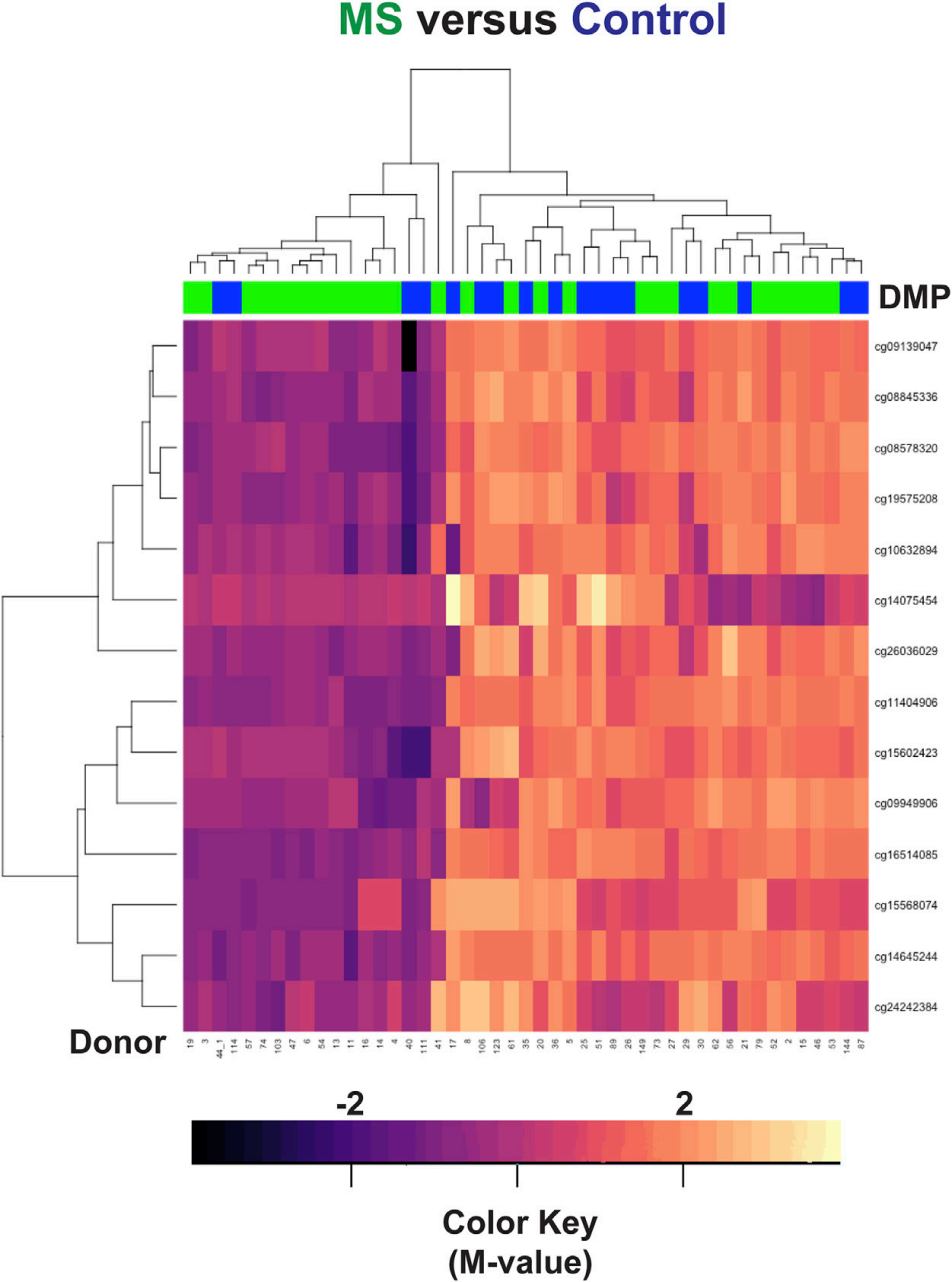
Hierarchal clustering analysis of the HLA-DRB1 region. Differences in methylation were observed at 14 CpG regions within HLA-DRB1 but did not necessarily correlate with disease state. In the heatmap, MS donors are designated by green, and controls by blue at the top of the heatmap. Blue/purple designates relative demethylation and orange/red increased methylation at a specific CpG site.

Differential methylation was observed at the remaining 14 loci but did not necessarily indicate disease state (Figure 3). Four out of 18 control samples were hypomethylated in this region, whereas 14 out of 29 MS samples were hypomethylated. These findings primarily reflect differences in HLA-DRB1 genotype in our patient population. Hypomethylation in this region (14 MS and 4 controls) likely signifies that they are HLA-DRB1*15:01 positive.

### Identification of gene-level biomarkers by DMR analysis

The next goal was to identify gene level differences between MS and controls. The DMRcate package was used to identify differentially methylated regions (DMR’s) (Peters et al., 2015). DMR’s contain multiple CpG loci that may be differentially methylated within a particular gene. This analysis increases the statistical power. As with the MD plots (Figure 1), the adjusted M values were used for this analysis and included corrections for disease modifying treatments.

**FIGURE 4:**
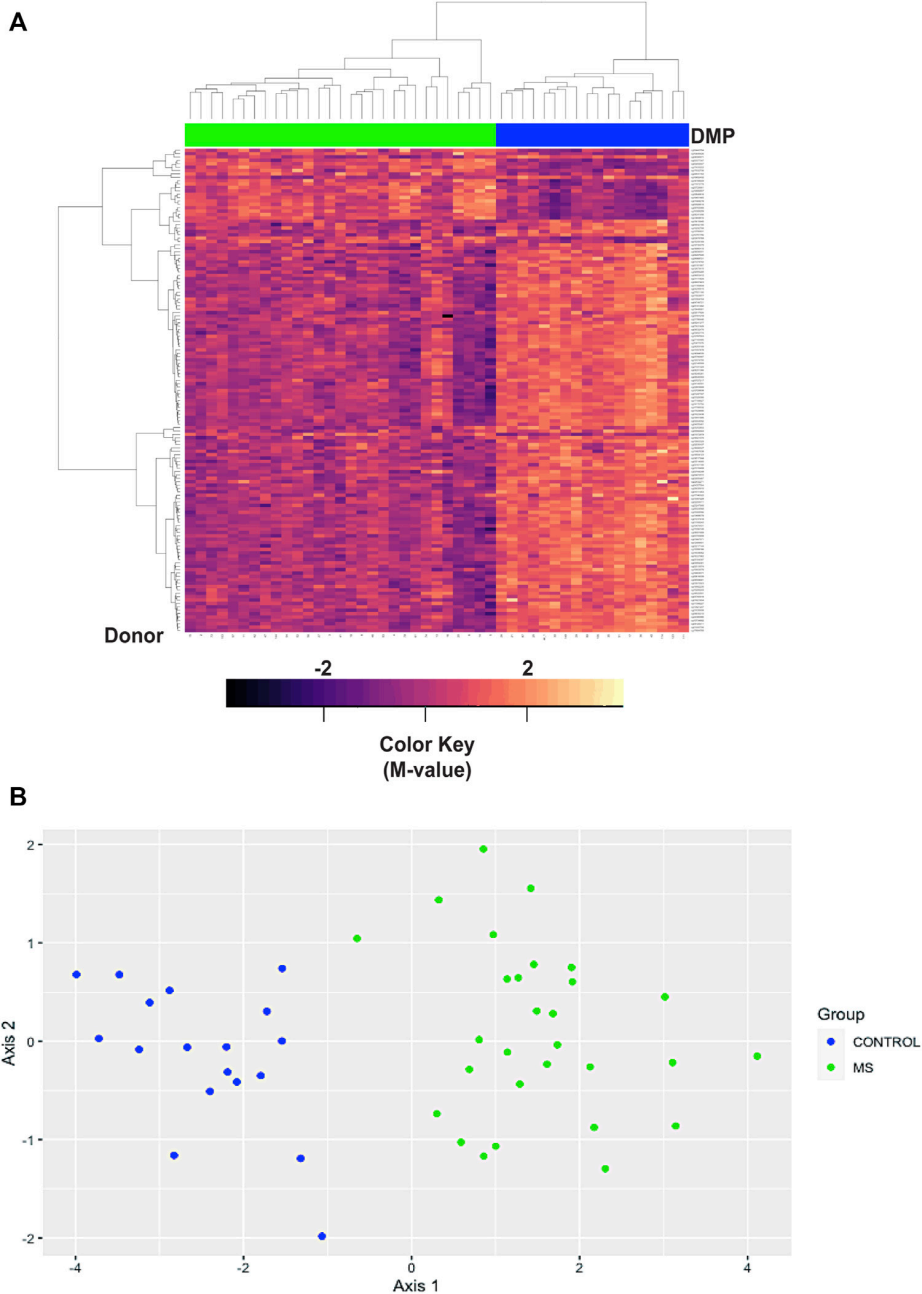
Hierarchal clustering and multi-dimensional scaling (MDS) analysis of differentially methylated regions associated with MS. **(A)** Hierarchal clustering analysis was performed on 147 differentially methylated loci within the top 10 differentially methylated regions (Supplementary Table S15). In the heatmap, MS donors are designated by green, and controls by blue at the top of the heatmap. Blue/purple designates relative demethylation and orange/red increased methylation at a specific CpG site. Gene regions included: ARID5B, BAZ2B, CDK2AP1, CLU, CTSZ, RAB34, RABGAP1, SFRP2, TNFSF12-TNFSF-13, and WBP1L. **(B)** MDS analysis was performed on the same data set and showed a goodness of fit (GOF) of 0.72.

We first analyzed differences in a comparison of all MS patients *versus* all Controls, irrespective of race or ethnicity. This analysis revealed 10,450 regions of interest (Table S14; FDR<1.93e-6, HMFDR ≤0.005). Using hierarchal clustering analysis, a subset of 147 DMP’s (Supplementary Table S15) within the top 10 DMR’s (FDR<1E-77, HMFDR≤1.15<e-6) was sufficient to differentiate MS from controls (Table 5; Figure 4A). Gene regions included: ARID5B, BAZ2B, CDK2AP1, CLU, CTSZ, RAB34, RABGAP1, SFRP2, TNFSF12-TNFSF-13, and WBP1L. These genes were not found to be differentially methylated in a comparison of all Black American donors *versus* all White donors (not shown). ARID5B, BAZ2B, RABGAP1, SFRP2, and WBP1L have not been previously associated with MS risk, and all are associated with neoplastic diseases and cellular proliferation.

**TABLE 5:** Top 10 differentially methylated regions (DMR) associated with multiple sclerosis.

DMR	Chromosome	Start	End	#CpGs	FDR (min smoothed)	HMFDR	Max difference M Value (MSvCon)	Mean difference M Value (MSvCon)	Overlapping genes
1	chr8	27467783	27470225	14	1.20E-136	2.43E-07	−0.05091	−0.03652	CLU
2	chr17	27044169	27045894	21	6.15E-111	1.79E-05	−0.06709	−0.03536	RAB34
3	chr9	1.26E+08	1.26E+08	14	1.89E-107	9.11E-07	−0.07037	−0.0436	RABGAP1
4	chr10	63807168	63809170	17	1.50E-94	3.11E-06	0.056923	0.040503	ARID5B
5	chr17	7460485	7462249	15	8.03E-94	3.34E-06	−0.05216	−0.02834	TNFSF12-TNFSF13
6	chr12	1.24E+08	1.24E+08	14	2.76E-90	1.35E-06	−0.06444	−0.03567	CDK2AP1
7	chr20	57581529	57583709	27	1.91E-89	1.58E-05	−0.0482	−0.0146	CTSZ
8	chr10	1.05E+08	1.05E+08	14	8.26E-82	8.14E-07	−0.04491	−0.02297	WBP1L
9	chr4	1.55E+08	1.55E+08	36	1.98E-81	1.97E-05	−0.04727	−0.01471	SFRP2
10	chr2	1.6E+08	1.6E+08	16	6.13E-78	1.15E-06	−0.07885	−0.03049	BAZ2B

DMR: differentially methylated region, CpG: 5′-cytosine-phosphate-guanine-3′, FDR: false detection rate, HMFDR: harmonic mean of individual CpG FDR’s.

In the hierarchal cluster analysis, approximately 86% of loci (123/143) demonstrated relative demethylation in the MS group as compared to controls. Relative demethylation at these DMR’s was observed for 9/10 of the gene regions (maximal and mean differences in M-values, Table 5). Taken together with the distribution of probes in the MD plot (Figure 1), these results suggested a tendency toward relative demethylation of DMR’s in the MS group compared to controls (Table 6).

**TABLE 6:** Pyrosequencing analysis.

Chromosome	Assay ID#	Position	Strand	Name	Gene	Region	CpG#
chr2	PM00685013	160473461	+	cg17503977	BAZ2B	TSS1500	3
chr9	PM00685139	125795935	+	cg14115756	RABGAP1	TSS1500	3
chr8	PM00683935	27469338	+	cg13488078	CLU	TSS1500	3
							
**Condition**	**BAZ2B**	**CLU**	**RABGAP1**				
	**%Methylation**	**%Methylation**	**%Methylation**				
**MS (n = 6)**	24.9 ± 2.3**	17.1 ± 1.5**	15.3 + 1.2*				
**Control (n = 4)**	39.6 ± 2.0**	25.6 ± 1.4**	21.2 + 1.4*				

*p = 0.004, **p < 0.001.

Multi-dimensional scaling (MDS) was also used to assess similarities in the DMR datasets of differential methylation based on disease state (MS *versus* controls, Figure 4B). As with the hierarchal cluster analysis, the 143 probes in the top 10 DMR were sufficient to differentiate MS from controls (Figure 4B). The goodness of fit (GOF) for this MDS analysis was 0.72.

As described for the analysis of the top DMP’s, we also performed mQTL analysis of the 143 probes within the top 10 DMR. There were 66 regions associated with mQTL (Supplementary Table S16) and 77 that were not (Supplementary Table S17). Hierarchal cluster analysis and MDS plots are shown in Supplementary Figures S3,S4. The GOF was 0.71 for those associated with mQTL and 0.74 for those not. These analyses also showed differentiation of MS from controls. Plots of the eigenvalues for the MDS plots are shown in Supplementary Figure S5.

### Identification of gene-level biomarkers by DMR analysis in racial and ethnic subgroups

**FIGURE 5:**
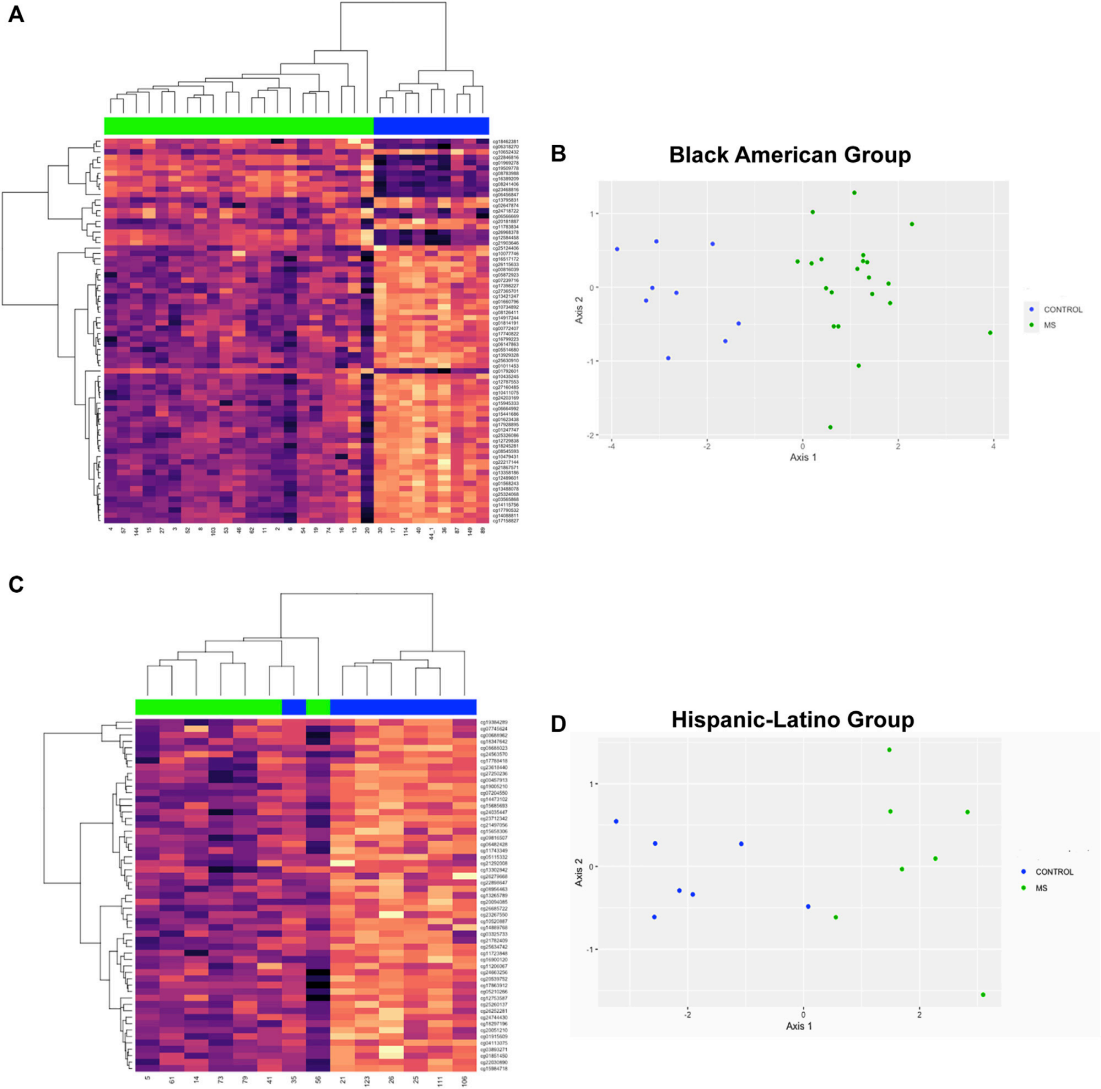
Hierarchal clustering and multi-dimensional scaling (MDS) analysis of differentially methylated regions associated with MS in racial and ethnic subgroups. **(A)** The top 10 DMR’s for the comparison of MS *versus* Controls for Black Americans included 6 regions that were present in the analysis of all donors (ARID5B, CDK2AP1, CLU, CTSZ, RABGAP1, and TNFSF12-TNFSF-13) and 4 other regions that reached statistical significance in the analysis of all donors but that were not in the top 10 regions (DUSP6, FOXI2, GPX6, and SPI2). 72 DMP’s (Supplementary Table S19) within the top 10 DMR’s were used for hierachal cluster analysis. **(B)** MDS plot showed a GOF = 0.81. **(C)** In the Hispanic-Latino subgroup, the top 10 DMR’s were: HOXD8, HPS4, KCNIP4, mir124-2, PTCHD4, PHYHIPL, RAB32, TREML2, UNC5, and WBSCR17. 55 probes within the top 10 DMR’s were used for hierarchal cluster analysis (Supplementary Table S21). One control outlier was observed in the MS cluster. **(D)**) MDS revealed a GOF = 0.75.

DMR analysis was also performed in racial and ethnic subgroups. In the comparison of MS *versus* Control in the Black American subgroup, 3127 regions of interest were identified (Supplementary Table S18, FDR<1.94e-5). The top 10 DMR’s included 6 regions that were present in the analysis of all donors (ARID5B, CDK2AP1, CLU, CTSZ, RABGAP1, and TNFSF12-TNFSF-13) and 4 other regions that reached statistical significance in the analysis of all donors but that were not in the top 10 regions (DUSP6, FOXI2, GPX6, and SPI2). The hierarchal cluster analysis for 72 DMP’s (Supplementary Table S19) within the top 10 DMR’s is shown in Figure 5A and the MDS plot in Figure 5B (GOF = 0.81).

In the Hispanic-Latino subgroup, 2285 regions of interest were identified (FDR<1.01e-10; Supplementary Table S20). The top 10 DMR’s were: HOXD8, HPS4, KCNIP4, mir124-2, PTCHD4, PHYHIPL, RAB32, TREML2, UNC5, and WBSCR17. In the hierarchal clustering analysis of this subgroup, 55 probes within the top 10 DMR’s was used (Supplementary Table S21). One control outlier was observed in the MS cluster (Figure 5C). However, MDS analysis showed differentiation between MS and controls (GOF = 0.75, Figure 5D).

### Confirmation of DMR results by pyrosequencing

**FIGURE 6:**
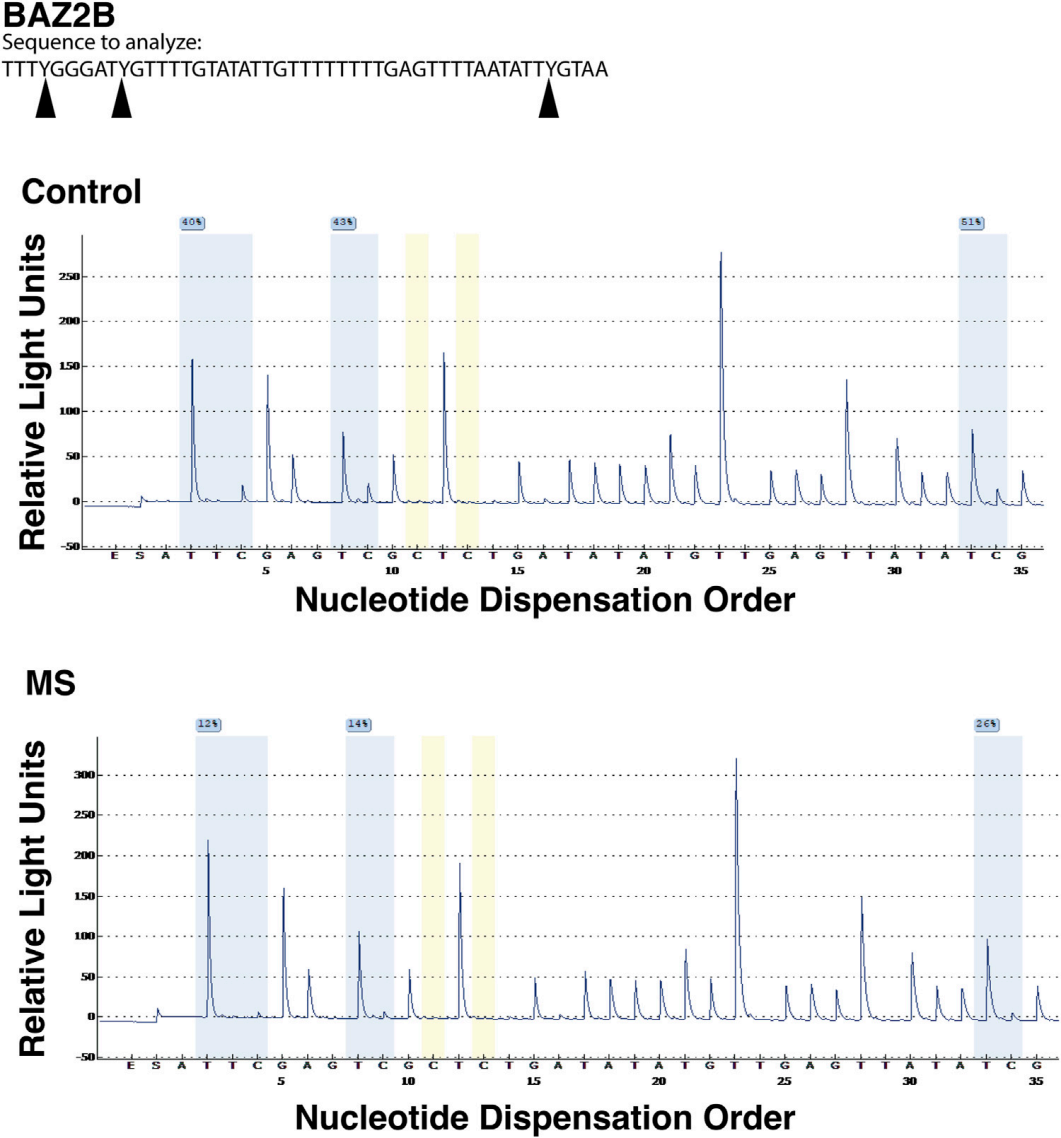
Pyrosequencing analysis of differentially methylated regions. Bead array data were confirmed by pyrosequencing following bisulfite conversion of the differentially methylated region of interest. In this representative analysis, the region of interest is in the promoter region (TSS1500) of BAZ2B gene (sequence is at the top of the figure). CpG regions are highlighted in blue, the regions utilized for bisulfite conversion controls are shown in yellow. For each of the 3 CpG regions analyzed, there was a relative reduction in the %methylation for the MS donor (bottom pyrogram) compared to the control (top).

Three DMR regions (BAZ2B, CLU, and RABGAP1) were selected for confirmation by pyrosequencing. The common feature of these regions is that they all contain multiple loci in close proximity that demonstrated relative demethylation in the TSS1500 region (1,500 bp upstream of the transcriptional start site) in the MS group. The regions and assay details are shown in Table 7. A representative example of the pyrosequencing analysis is shown for the BAZ2B gene region in Figure 6. The sequences of interest for the 3 genes (BAZ2B, CLU, and RABGAP1) contain 3 CpG sites, and data were pooled for analysis of each of the differentially methylated regions. There was a statistically significant reduction in relative percent methylation at the CpG sites within each of the analyzed regions for BAZ2B (*p* < 0.0001), CLU (*p* < 0.0001), and RABGAP1 (*p* = 0.0004) (Table 7).

**TABLE 7:** KEGG pathway analysis of differential methylation with dimethyl fumarate treatment.

KEGG pathway	Description	N (loci)	DE	P.DE	FDR
path:hsa04060	Cytokine receptor interaction	280	85	0.00043	0.0756
path:hsa04360	Axon guidance	175	90	0.00052	0.0756
path:hsa04520	Adherens junction	70	41	0.00067	0.0756
path:hsa04062	Chemokine signaling pathway	189	79	0.00088	0.0756

DE, discrete elements (genes); FDR, false detection rate.

### Identification of gene-level differentially methylated regions associated with dimethyl fumarate treatment

Analysis was also performed in the MS subgroup (n = 29) to compare differential methylation of those patients treated with dimethyl fumarate (n = 12) *versus* all other individuals with MS (n = 17, 8 on glatiramer acetate, 6 on ocrelizumab, 1 on beta-interferon, and 1 untreated). Probes were filtered as described above, and adjustments were made for gender, age, race, and latent variables. However, unlike the prior analyses, disease modifying treatments were listed as a factor to be preserved. This analysis showed 1485 DMP’s (Supplementary Table S22) with an FDR<0.01 (p < 2E-5) and 12,915 with an FDR<0.1 (*p* < 0.01). KEGG pathway analysis of those probes identified possible associations with cytokine receptor interactions, adherens junction regulation, chemokine signaling, and axonal guidance (Table 8).

**TABLE 8:** Top 10 differentially methylated regions (DMR) associated with dimethyl fumarate treatment.

DMR	Chromosome	Start	End	#CpGs	FDR (min smoothed)	HMFDR	Max difference M Value (DMFvOther)	Mean difference M Value (DMFvOther)	Overlapping genes
1	chr22	44463707	44465038	10	2.57E-64	0.001067	−0.07046	−0.0306	PARVB
2	chr17	27044169	27045894	21	2.19E-55	0.01804	−0.07319	−0.03276	RAB34
3	chr10	1.05E+08	1.05E+08	14	5.76E-53	0.003279	−0.04699	−0.01822	WBP1L
4	chr3	1.12E+08	1.12E+08	15	1.42E-52	0.007249	−0.04847	−0.02573	TAGLN3
5	chr22	44568203	44568812	9	9.40E-51	0.004387	−0.05	−0.03259	PARVG
6	chr5	1.77E+08	1.77E+08	13	2.84E-50	0.003224	−0.04945	−0.02524	DOK3
7	chr12	51403056	51403966	6	5.95E-48	0.000422	−0.05586	−0.02471	SLC11A2
8	chr7	1094263	1096387	12	2.54E-46	0.004215	−0.04168	−0.02055	GPR146
9	chr8	27467783	27469673	13	1.19E-44	0.0076	−0.04168	−0.02666	CLU
10	chr3	33700962	33701707	9	2.44E-40	0.003399	−0.03639	−0.02407	CLASP2

*12 patients on dimethyl fumarate, 8 on glatiramer, 6 on ocrelizumab, 1 on interferon, and 1 on natalizumab.

DMR, differentially methylated region; CpG, 5′-cytosine-phosphate-guanine-3′; FDR, false detection rate; DMF, dimethyl fumarate; HMFDR, harmonic mean of individual CpG FDR’s.

**FIGURE 7:**
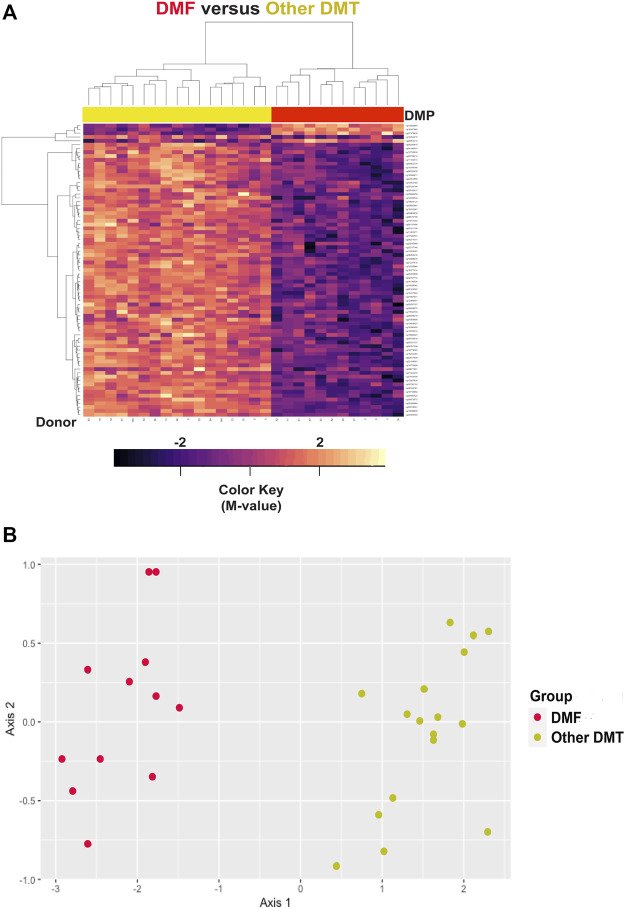
Hierarchal clustering and multi-dimensional scaling (MDS) analysis of differentially methylated regions associated with dimethyl fumarate treatment. **(A)** Heatmap is shown for hierarchal clustering analysis performed on differentially methylated loci within 77 differentially methylated loci within the top 10 differentially methylated regions (DMR) (Supplementary Table S23). The gene regions are: CLASP2, CLU, DOK3, GPR146, PARVB, PARVG, RAB34, SLC11A2, TAGLN3, and WBP1L. In the heatmap, dimethyl fumarate treatment is designated by red (n = 12), and controls by yellow at the top of the heatmap (n = 17). Blue/purple designates relative demethylation and orange/red increased methylation at a specific CpG site. **(B)** MDS analysis showed a goodness of fit of 0.81.

Hierarchal cluster and MDS analysis were performed on 77 DMP’s (Supplementary Table S23) within the top 10 DMR’s (FDR<2.5E-40). These gene regions included: CLASP2, CLU, DOK3, GPR146, PARVB, PARVG, RAB34, SLC11A2, TAGLN3, and WBP1L. Four of these genes (CLASP2, PARVB, PARVG, and TAGLN3) regulate the cytoskeleton, and three of them were also identified in the top 10 DMR’s for the MS *versus* controls comparison (CLU, RAB34, and WBP1L). As shown in Table 8 (maximal and mean differences between groups), all these regions showed relative demethylation in the DMF group as compared to those not on DMF. As shown in the heatmap of the hierarchal clustering analysis (Figure 7A) and in the MDS plot (Figure 7B), analysis of these regions was sufficient to distinguish those individuals on dimethyl fumarate *versus* all other MS patients (GOF = 0.81 for the MDS analysis).

## Discussion

This epigenome-wide association study demonstrated unique patterns of global and gene level differential DNA methylation in our MS patient population. To our knowledge, this study is the first to focus on differential DNA methylation in an underrepresented population of MS patients in the United States. Notable findings included distinct global patterns of differential demethylation in MS, a preferential association with enhancer regions rather than promoters, and identification of novel gene level biomarkers associated with MS and disease modifying treatments. There was a tendency for many of the differentially methylated regions to demonstrate relative demethylation in MS. In addition, pathway analysis suggested possible associations of epigenetic biomarkers of cellular differentiation, Shigellosis, and amoebiasis in our patient cohort.

The most notable observation in the analysis of global differential DNA methylation in MS was a tendency toward demethylation of probe regions that demonstrated relatively low levels of methylation across all donors. Although exceptions exist, there is a tendency for CpG islands to be hypomethylated in normal cells and hypermethylated in neoplastic cells (Bird and Wolffe, 1999; Baylin et al., 2001; Michal and Wojtas, 2019). Some cancers, such as high-grade pediatric gliomas, are associated with DNA hypomethylation (Bender et al., 2013). Many prior studies suggested that hypomethylated regions in a variety of cancers occur at introns and intergenic regions (Wilson et al., 2007). More recent studies in chronic lymphocytic leukemia and other hematologic malignancies revealed an association of hypomethylation of promoter regions (Upchurch et al., 2016). This observation may be most relevant to this study because we examined whole blood methylomics. Taken together with our pathway analysis results, these data suggest a possible relationship between the pathogenesis of MS in some patient populations and hematological malignancies.

Consistent with those results, we also observed increased representation of enhancer regions and decreased frequency of associations with promoters among differentially methylated probe regions. These differential DNA methylation patterns of enhancers have been associated with neoplastic transformation, metastasis of solid tumors, and myelodysplastic diseases (Bell et al., 2016; Ordonez et al., 2019). In addition, gene level analysis suggested a pattern of hypomethylation of a subset of genes in putative promoter regions (TSS1500). For example, one of the genes analyzed by pyrosequencing analysis, BAZ2B, regulates chromatin structure and hematopoietic cell development (Arumugam et al., 2020). These results will need to be confirmed in larger data sets.

Pathway analysis revealed unexpected associations with gastrointestinal infections due to bacteria and parasites but not with viral infection. The specific pathways identified were for Shigellosis, amoebiasis, and bacterial invasion of epithelia. Shigellosis may be particularly relevant to our patient population because frequent outbreaks have been identified in Chicago (Jones et al., 2006). In addition, one prior genetic study suggested an association of the Shigellosis pathway with MS and Crohn’s disease (Restrepo et al., 2016). The possible association with amoebiasis may be relevant to our Hispanic-Latino population who have emigrated from Mexico and Central America and those who have relocated from Puerto Rico. Although these findings need to be examined in greater detail, they suggest that prior bacterial and parasitic gastrointestinal infections may be more relevant to MS susceptibility in our patient cohort than prior viral infections such as Epstein Barr Virus (Bjornevik et al., 2022).

Prior studies of differential DNA methylation in MS have focused primarily on patients of Northern European ancestry (Kulakova et al., 2016; Kular et al., 2018; Souren et al., 2019; Kiselev et al., 2021). A prior study demonstrated relative hypomethylation of the HLA-DRB1 region in MS patients (Kular et al., 2018). We examined this region in our patient cohort and observed a subset of individuals that had hypomethylation of this region, but it did not correlate with disease state. Although these results may suggest that epigenetic biomarkers of MS may differ between racial and ethnic groups, these data likely reflect differences between HLA-DRB1 haplotypes in our patient population. Several other recent studies of differential methylation in MS have focused on specific immune cell populations. These include analyses of CD4^+^ T lymphocytes (Ewing et al., 2019; Roostaei et al., 2021), CD8^+^ T lymphocytes (Li et al., 2017; Deng et al., 2019; Ewing et al., 2019), monocytes (Ewing et al., 2019; Diniz et al., 2021), and CD19^+^ B lymphocytes (Maltby et al., 2018a).

For gene level analysis of differential methylation associated with MS in our patient cohort, we focused on the top 10 DMR’s: ARID5B, BAZ2B, SFRP2, WBP1L, CDK2AP1, CLU, CTSZ, RAB34, RABGAP1, and TNFSF12/TNFSF13. One of these gene regions, CTSZ, was previously reported to be hypomethylated in MS in post-mortem brain tissue (Huynh et al., 2014) and to also be associated with risk of systemic sclerosis (Zhu et al., 2018). This latter observation may be of relevance to the current study because Black Americans have a higher incidence of systemic sclerosis and are at increased risk of a more severe disease course (Steen et al., 2012; Steen et al., 1963-1982). One potential mechanism for CTSZ to mediate pro-inflammatory effects is through increased interleukin 1β secretion by antigen presenting cells (Allan et al., 2017).

Although the other regions have not been shown to be differentially methylated in prior studies, some of these genes have been associated with MS in genomic, transcriptomic, and proteomic studies. For example, CDK2AP1*,* a cell cycle regulator, was previously identified as an MS risk allele that correlated with reduced RNA expression in lymphoblast cells and peripheral blood mononuclear cells (PBMC’s) (IMSGC, 2010). It was also identified as a susceptibility gene for MS in the genomic map IMSGC study (Supplementary Table S13) (International, 2019). Other studies demonstrated increased RNA expression of CLU*,* a calcium binding protein, in peripheral blood from MS patients (Razia et al., 2022) and increased protein levels in cerebrospinal fluid (van Luijn et al., 2016). RAB34 (Liu et al., 2022) and the TNFSF12-TNFSF13 (Krumbholz et al., 2008) have also been associated with increased MS risk.

The more novel findings in this study were in genes (ARID5B, BAZ2B, RABGAP1, SFRP2, and WBP1L) that are associated with cancer risk and cellular differentiation. For example, ARID5B*,* has been associated with leukemia (Treviño et al., 2009), prostate cancer (Davalieva et al., 2015), gastric cancer (Lim et al., 2014), and endometrial carcinoma (Kandoth et al., 2013). As discussed above, BAZ2B regulates chromosome structure, hematopoietic cell development, and reprogramming of pluripotent stem cells (Arumugam et al., 2020). RABGAP1 regulates mitosis, cell migration, and mTOR signaling (Oh et al., 2022). SFRP2 is a tumor suppressor protein that can induce cell apoptosis, and differential methylation of its promoter region has been associated with leukemias and renal cancer (Jost et al., 2008; Konac et al., 2013; Li and Luo, 2018). WBP1L regulates proapoptotic pathways in myeloid cell leukemia (Morishima et al., 2011). Differential methylation of these 5 gene regions further suggests an association between regulation of neoplasia and cellular proliferation in our patient population. These epigenetic determinants may be mediated by environmental exposures that increase the risk of some cancers and autoimmune diseases (Dor and Cedar, 2018).

This study also demonstrated the feasibility of performing parallel analyses to detect associations with MS and disease modifying treatments. This analysis is important because it may inform treatment decisions based on biomarkers of medication responders and non-responders. In addition, in real world practice, monitoring of biomarkers over the patient’s disease course requires approaches that allow ongoing disease modifying treatment to continue. In this study, we focused on differential DNA methylation associated with dimethyl fumarate treatment because that group represented the largest treatment cohort in this pilot study. Analysis of probes within the top DMR’s demonstrated relative hypomethylation of these loci in the dimethyl fumarate group. In a prior study of the effect of dimethyl fumarate in CD4 T lymphocytes, four differentially methylated loci were observed, SNORD1A, SHTN1, MZB1 and TNF (Maltby et al., 2018b). We observed differential methylation of SHTN1 in our comparison of the top 10,000 DMP’s for the MS *versus* Control comparison (Supplementary Tables S5–S7), but not in the comparison of dimethyl fumarate *versus* other treatments. One common feature is that differential methylation of TNF was observed in that study and in our own. Another study assessed differential methylation in monocytes and CD4 T lymphocytes prior to initiation of dimethyl fumarate and following treatment (Carlstrom et al., 2019). A potentially important observation between our study and theirs is an association of differential methylation with cytokine pathways, including IL6 and IL17 regulated signaling. In a study that focused only on global patterns of differential methylation, INFβ treatment significantly reduced global methylation in monocytes but not in lymphocyte of MS patients (Diniz et al., 2021). Additional studies are required in the future to assess potential biomarkers associated with other treatments.

This study has several limitations. One is that it is a pilot study on a limited number of patients from our clinical practice. Further studies are required with larger numbers of patients. In addition, some of the findings may reflect regional differences due to unique environmental exposures, and additional studies are necessary in other patient cohorts. However, even if some of the differential DNA methylation are due to regional differences, it is important to identify those biomarkers associated with geographic location to better understand disease heterogeneity on a national level. Another limitation is that this study focused on whole blood methylomics, and many relevant methylation differences in the CNS may be missed. However, pathway and gene level analysis, including CLU and RAB34, revealed associations with axonal regulatory pathways and gene regions identified in prior studies of CNS tissue (Liu et al., 2022) and CSF (van Luijn et al., 2016). Our results are consistent with a prior study that demonstrated the feasibility of detecting CNS relevant differential methylation in peripheral blood samples. That study suggested that analysis of peripheral blood samples can detect approximately 20%–30% of differential methylation observed in live brain tissue (Braun et al., 2019). In addition, an important feature of using whole blood methylomics is that it can be assessed using a minimally invasive approach, which is important for longitudinal assessments in real world clinical practice. Integrated analysis with single cell approaches such as RNAseq also can be used to assess the relevance of differential DNA methylation in specific immune cell subtypes. An additional limitation of the current study is that HLA typing and genome-wide genotyping were not performed. Integrated analysis of these data with methylomics will need to be performed in future studies of our patient cohort. In addition, it will be important to analyze the associations of differentially methylated regions with environmental and social determinants of health, particularly for those regions not associated with SNP, eQTL, or mQTL regions.

Despite the limitations of this study, the results allow us to develop a working model to postulate possible pathobiological differences of MS susceptibility in select populations. Overall, the results suggest that DNA hypomethylation of many gene regions previously associated with neoplastic regulation are associated with MS susceptibility in Black and Hispanic American patients in our cohort. Additional studies are required to assess the relevance of these findings to the proliferation, invasiveness, and pathogenicity of specific immune cell populations.

## Methods

### Subjects

This was a cross-sectional, case-control study. 29 subjects with multiple sclerosis (MS) and 18 controls with non-inflammatory neurological disease were enrolled (Supplementary Tables S1). All subjects were followed at the University of Illinois-Neurosciences Center and were enrolled in the University of Illinois at Chicago (UIC) Neuroimmunology Biobank between August, 2018 and October, 2019. The UIC Neuroimmunology Biobank is approved by the Institutional Review Board (IRB) of the University of Illinois College of Medicine. All subjects provided informed written consent at enrollment.

### Inclusion and exclusion criteria

MS donors met the following criteria: 1) age between 18 and 80 years at the time of enrollment, 2) a diagnosis of relapsing-remitting MS (RRMS) based on the McDonald criteria 2017 (Thompson et al., 2018), 3) no history of relapse(s) 30 days prior to the sample collection, 4) no history of receiving steroids within 30 days prior to the sample date, 5) no MRI activity within 30 days prior to the sample collection date (if MRI available), and 6) availability of the clinical data at the time of sample collection. The control group met these criteria: 1) between 18 and 80 years at the time of sampling, 2) presentation with a neurological complaint other than a neuro-inflammatory or neurodegenerative disorder, 3) no history of a recent ischemic stroke within the 6 months prior to the sample date, 4) no history of a systemic autoimmune disease, and 5) ambulatory without assistance at the time of sampling. Exclusion criteria for both MS and control subjects were: 1) failing to meet the inclusion criteria or 2) being on an immunomodulating or immunosuppressant agent other than the disease modifying treatments for MS within 6 months prior to the sample date.

### Whole blood methylomics of genomic DNA

Whole blood genomic DNA (gDNA) was isolated from whole blood using EZ1 Advanced XL automated instrument (Qiagen Cat. No. 9001875) using EZ1&2 DNA blood 350 ul kit (Qiagen Cat. No.951054). Infinium MethylationEPIC bead arrays (Illumina) were utilized to characterize whole blood genomic DNA (gDNA) methylation of MS patients and controls Samples were randomized on the chip. All samples had very high CpG detection rates, and, therefore, none needed to be removed from the analysis.

### Normalization and filtering

Analysis was performed in R software (version 4.0.3). The session information and packages utilized are shown in Supplementary Table S24. Data were normalized using the preprocessQuantile function from the minfi R package (Aryee et al., 2014). Probes were filtered and removed from analysis for low detection value in one or more samples (4,726 probes), sex differences (19,072 probes on the X and Y chromosomes), CpG sites associated with known SNP’s (single nucleotide polymorphisms; 28,567 probes), and probe cross-reactivity (24,690 probes) (Chen et al., 2013). 788,804 probes remained for further analysis.

### Linear model of differential methylation

Using the lmFit and eBayes functions from the limma package (Ritchie et al., 2015), preliminary models were fit as:

Y=Group+Race+Gender+Age

For all 47 samples, and:

Y=Group+Gender+Age

for the 30 Black American samples, and separately in the 14 Hispanic-Latino samples, where Y is the M-value indicating the degree of methylation at a given CpG, and Group indicates the MS or control group.

Removal of Unwanted Variation (Jacob et al., 2016) was used to identify any latent variables that should be included in analysis. The method of Buja and Eyuboglu (Buja and Eyuboglu, 1992) was implemented in the num.sv function of the sva package (Leek and Storey, 2007) indicated that 6 latent variables should be included in the model with all 47 samples, 4 latent variables should be included in the model with the 30 Black American samples, and 3 latent variables should be included in the model with the 14 Hispanic-Latino samples. Negative control probes were selected as those that had a *p*-value >0.5 for every effect in each of the respective above models, which yielded 27413 probes for the model with all 47 samples, 80572 probes for the model with the 30 Black American samples, and 78369 probes for the model with the 14 Hispanic-Latino samples. The iterateRUV function from the RUVnormalize package (Jacob et al., 2016) was then used to estimate latent variables under default parameters. There was no obvious relationship between the latent variables and demographic cofactors.

Models were then re-run as:

Y=Group+Race+Gender+Age+W

For all 47 samples, and:

Y=Group+Gender+Age+W

for the 30 Black American samples, and separately for the 14 Hispanic-Latino samples, where W represents the matrix of latent variables estimated for the respective model.

### Adjustment of M-values for covariates, including disease modifying treatments

The RUVnormalize R package (Jacob et al., 2016) was used to estimate unknown covariates in the dataset, some of which corresponded to disease modifying treatments. The removeBatchEffects function from the limma package (Ritchie et al., 2015) was used to adjust M-values. Group, race, and intercept were listed as factors to be preserved. Slide was indicated as a batch effect. Gender, age, and latent variables were indicated as covariates to be adjusted for. Data were analyzed in the presence and absence of adjustments for disease modifying treatments as a covariate. Adjusted M-values were used to draw Mean Difference (MD) plots using the glMDPlot function in the Glimma package (Su et al., 2017). These values were also used for analysis of DMP’s within DMR’s.

### Analysis of differentially methylated regions

The DMRcate package was used to identify differentially methylated regions (DMR’s) based on the *p*-values used to detect differentially methylated probes (DMP’s) (Peters et al., 2015). For each of the three models used for detecting DMP’s, an FDR threshold was determined at which probes with a *p*-value of 0.001 or lower would be captured. The dmrcate function was run with lambda = 1,000 and C = 2.

### KEGG pathway and cell composition analysis

The gometh function of missMethyl was used to identify enriched KEGG terms among differentially methylated genes (Phipson et al., 2016). Cell composition analysis was performed using the CellCounts2 function in the FlowSorted. Blood.EPIC package in R software (Salas et al., 2018).

### Pyrosequencing

Bisulfite conversion of gDNA was performed (EpiTect Bisulfide Kit (Qiagen Cat. No. 59104), and regions of interest were amplified by PCR (Qiagen Pyromark Custom Assays, Qiagen PyroMark kit Cat. No. 978703) using a QuantStudio 3 real time PCR system. Pyrosequencing and analysis were performed at the Stanford University School of Medicine’s Beckman Center for Molecular and Genetic Medicine. The regions analyzed and assay numbers are shown in Table 4. Data were analyzed in SPSS (version 28, IBM).

## Data Availability

The data presented in this study was deposited in the GEO repository, accession number GSE219293.
