# Supplementary material for: Differential DNA methylation associated with multiple sclerosis and disease modifying treatments in an underrepresented minority population
Source: Front Genet. 2023 Jan 4;13:1058817. doi: 10.3389/fgene.2022.1058817 (PMC9845287; doi:10.3389/fgene.2022.1058817)
Supplement: Supplementary file 1 [file DataSheet2.PDF]

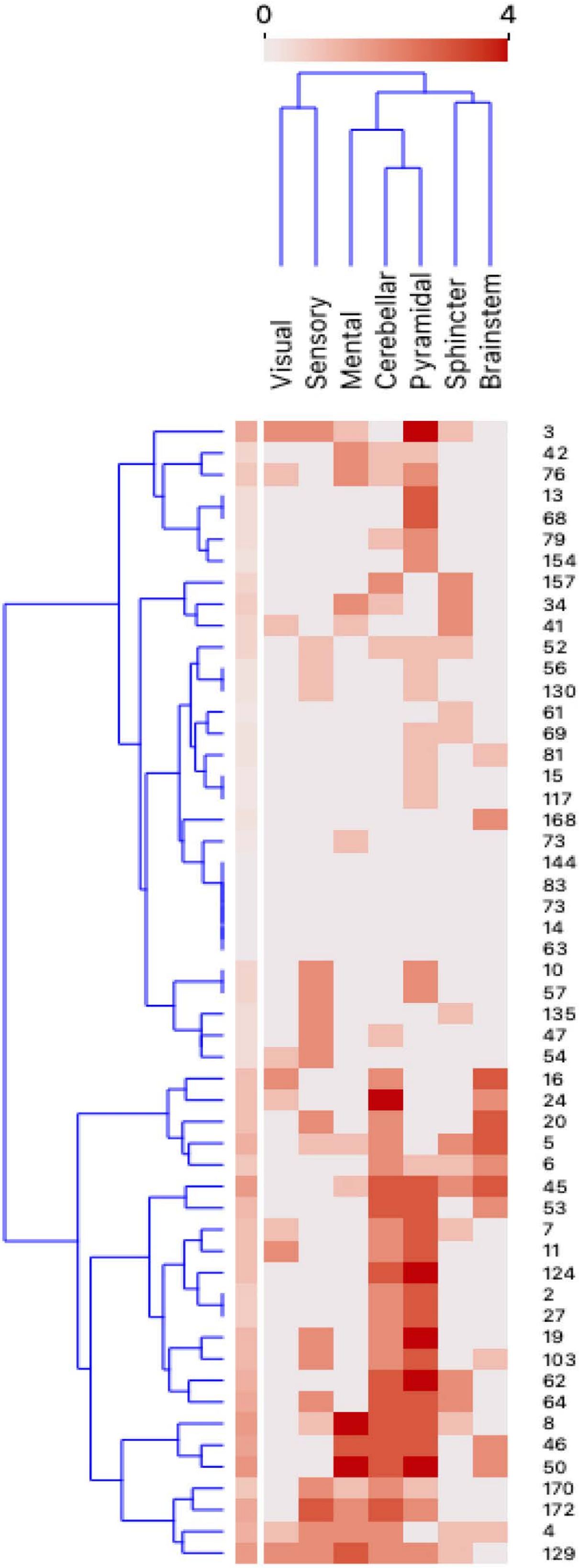

Figure S1. Assessment of clinical variation. The heatmap shows the clinical variation in our MS patient cohort using Functional Systems Scores. For more detailed deep clinical phenotyping of these patients, network analysis was performed on a larger number of patients from the same cohort (see reference 13).

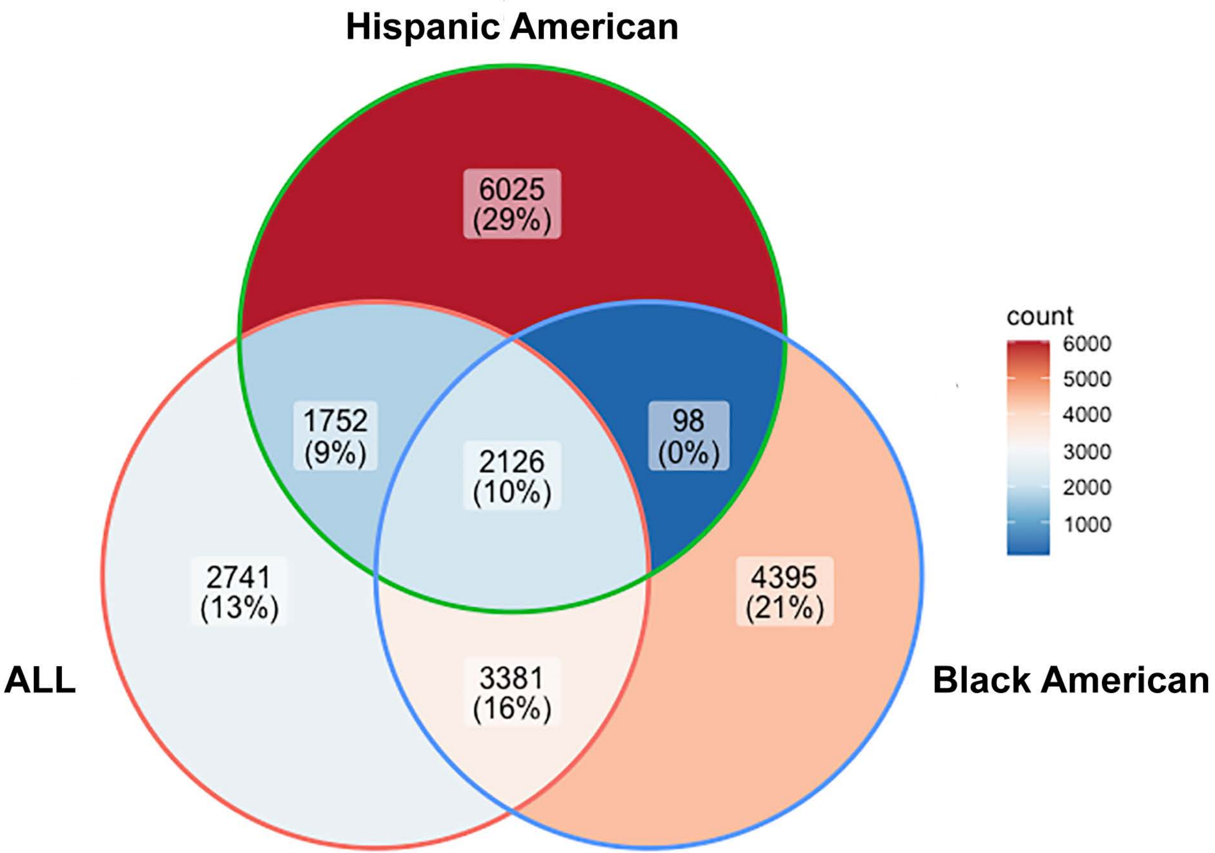

Figure S2. Venn diagram of the top 10,000 DMP's for each MS versus Control comparator groups. DMP tables are available in Figures S5-S9.

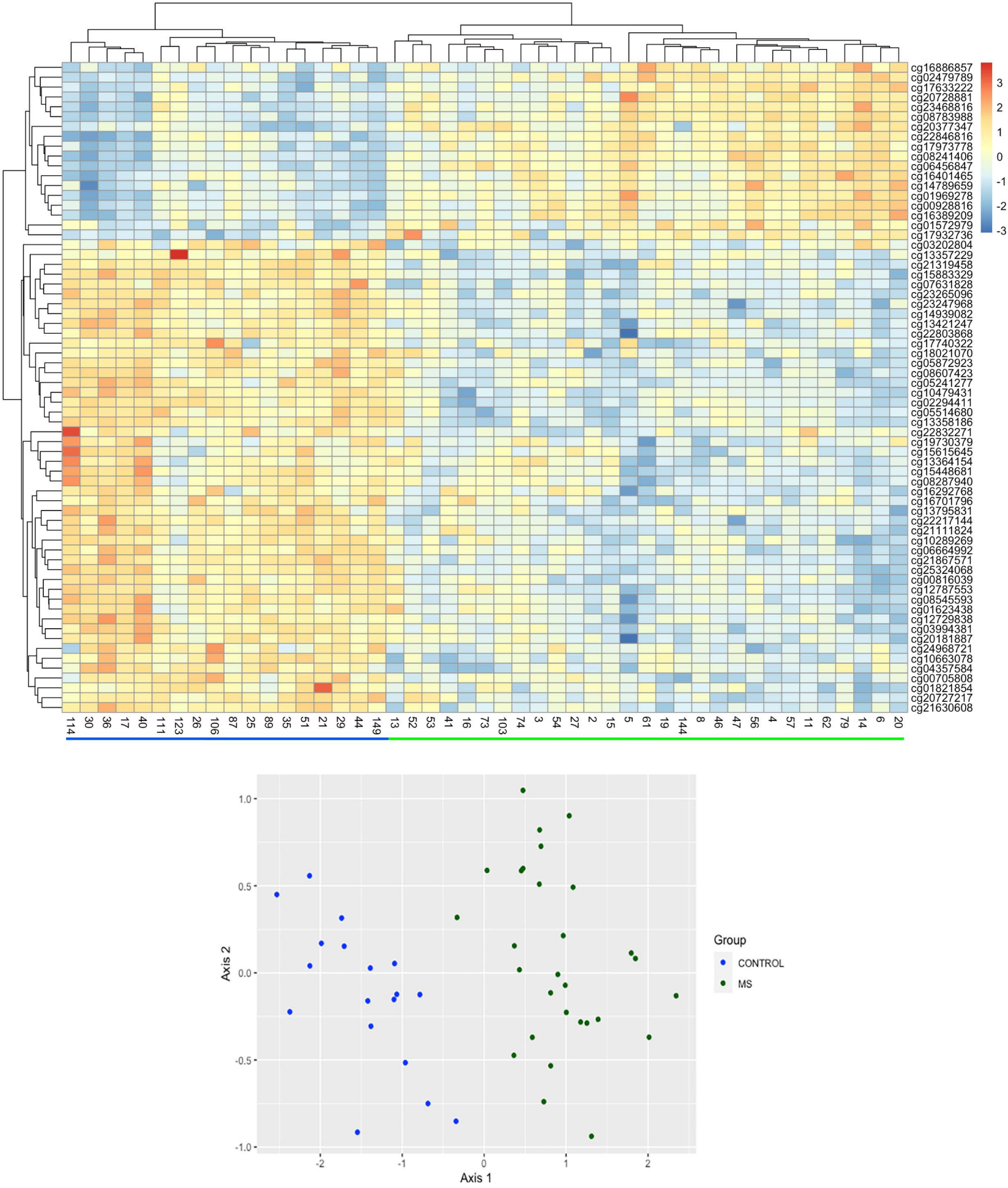

Figure S3. Hierarchal cluster analysis and MDS plot of probes in the top 10 DMR associated mQTL. The Pheatmap package was used to generate the heatmap (top). Probes are on the vertical acccess and donor ID's on the horizontal. MS patients are underlined in green and controls blue. MS and Controls separated in 2 major clusters. For the MDS plot, the GOF was 0.71, and the eigenvalues for the first 5 dimensions were: 78, 11, 4.4, 3.8, and 3.4.

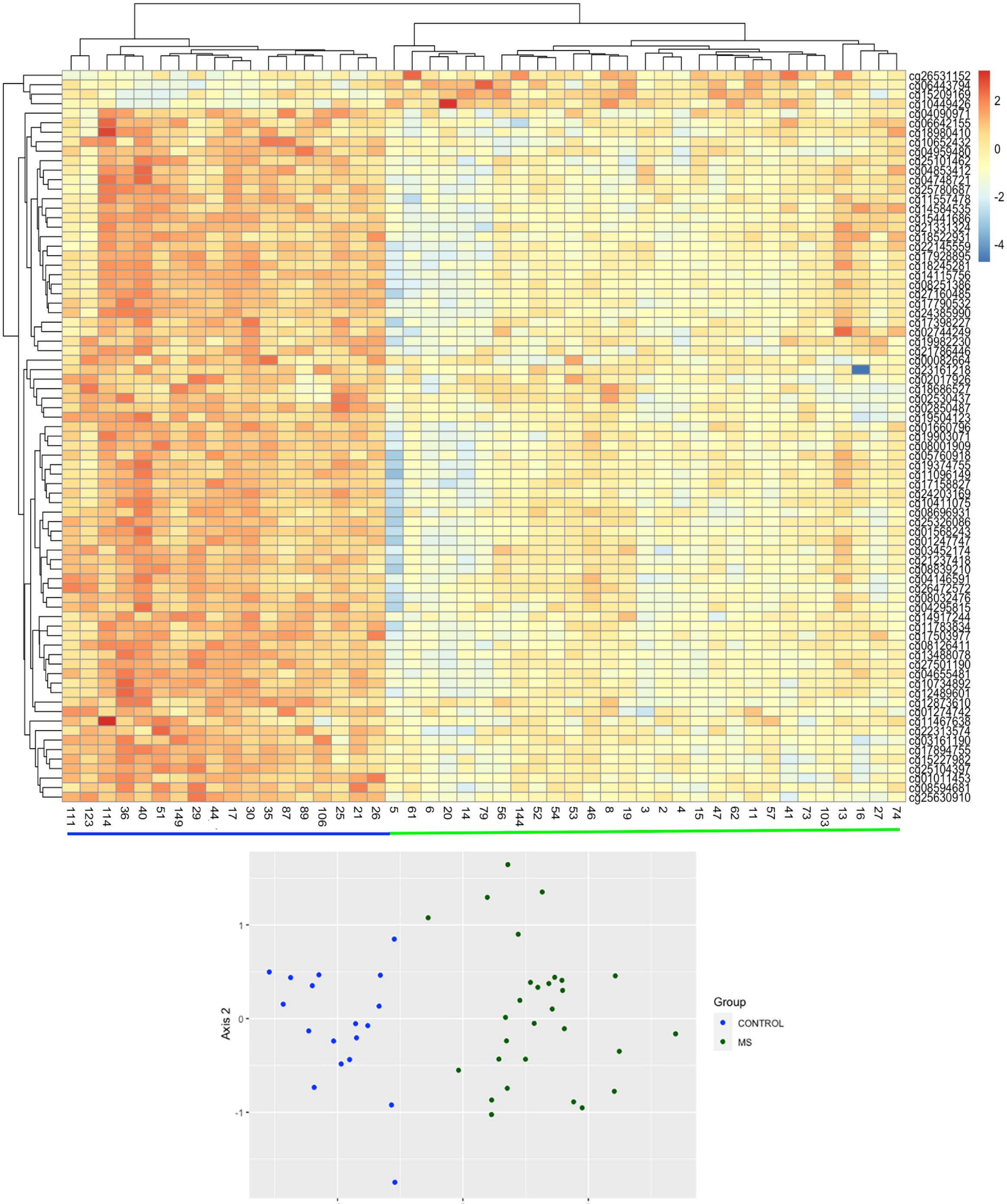

Figure S4. Hierarchal cluster analysis and MDS plot of probes in the top 10 DMR not associated mQTL. The Pheatmap package was used to generate the heatmap (top). Probes are on the vertical acccess and donor ID's on the horizontal. MS patients are underlined in green and controls blue. MS and Controls separated in 2 major clusters. For the MDS plot, the GOF was 0.74, and the eigenvalues for the first 5 dimensions were: 136, 22, 7.2, 6.1, and 4.8**.**

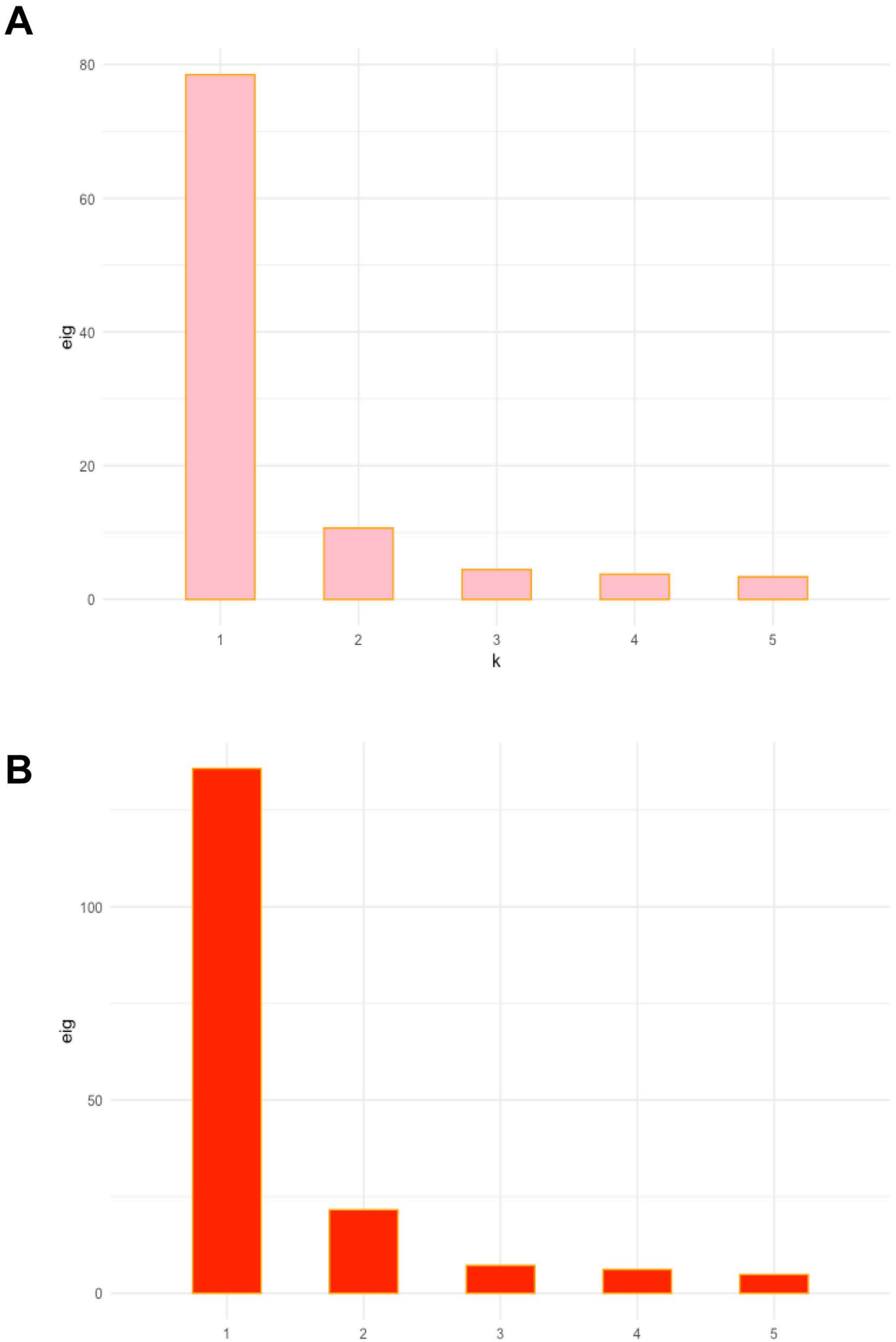

Figure S5. Eigenvalue plots. A.) DMR 10 probes associated with mQTL (Figure S2). The eigenvalues for the first 5 dimensions were: 78, 11, 4.4, 3.8, and 3.4. B.) DMR10 probes not associated with mQTL (Figure S3). Eigenvalues were: 136, 22, 7.2, 6.1, and 4.8.
