# Supplementary material for: Differential DNA methylation associated with multiple sclerosis and disease modifying treatments in an underrepresented minority population
Source: Front Genet. 2023 Jan 4;13:1058817. doi: 10.3389/fgene.2022.1058817 (PMC9845287; doi:10.3389/fgene.2022.1058817)
Supplement: Supplementary file 3 [file DataSheet1.PDF]

Table S1: Session Information and R Packages

```
## R version 4.0.3 (2020-10-10)
## Platform: x86_64-w64-mingw32/x64 (64-bit)
## Running under: Windows 10 x64 (build 19042)
##
## Matrix products: default
##
## locale:
## [1] LC_COLLATE=English_United States.1252
## [2] LC_CTYPE=English_United States.1252
## [3] LC_MONETARY=English_United States.1252
## [4] LC_NUMERIC=C
## [5] LC_TIME=English_United States.1252
##
## attached base packages:
##  [1] grid      stats4    parallel  stats     graphics  grDevices utils
##  [8] datasets  methods   base
##
## other attached packages:
##  [1] DMRcatedata_2.8.2
##  [2] spams_2.6
##  [3] Matrix_1.2-18
##  [4] lattice_0.20-41
##  [5] gridExtra_2.3
##  [6] FlowSorted.Blood.EPIC_1.8.0
##  [7] ExperimentHub_1.16.0
##  [8] AnnotationHub_2.22.0
##  [9] BiocFileCache_1.14.0
## [10] dbplyr_2.1.0
## [11] quadprog_1.5-8
## [12] RUVnormalize_1.24.0
## [13] ggplot2_3.3.3
## [14] viridis_0.5.1
## [15] viridisLite_0.3.0
## [16] gplots_3.1.1
```

Table S1: Session Information and R Packages

```
## [17] org.Hs.eg.db_3.12.0
## [18] AnnotationDbi_1.52.0
## [19] sva_3.38.0
## [20] BiocParallel_1.24.1
## [21] genefilter_1.72.1
## [22] mgcv_1.8-33
## [23] nlme_3.1-149
## [24] Glimma_2.0.0
## [25] stringr_1.4.0
## [26] DMRcate_2.4.1
## [27] Gviz_1.34.0
## [28] missMethyl_1.24.0
## [29] IlluminaHumanMethylation450kanno.ilmn12.hg19_0.6.0
## [30] RColorBrewer_1.1-2
## [31] IlluminaHumanMethylationEPICmanifest_0.3.0
## [32] IlluminaHumanMethylationEPICanno.ilm10b4.hg19_0.6.0
## [33] minfi_1.36.0
## [34] bumphunter_1.32.0
## [35] locfit_1.5-9.4
## [36] iterators_1.0.13
## [37] foreach_1.5.1
## [38] Biostrings_2.58.0
## [39] XVector_0.30.0
## [40] SummarizedExperiment_1.20.0
## [41] Biobase_2.50.0
## [42] MatrixGenerics_1.2.1
## [43] matrixStats_0.58.0
## [44] GenomicRanges_1.42.0
## [45] GenomeInfoDb_1.26.2
## [46] IRanges_2.24.1
## [47] S4Vectors_0.28.1
## [48] BiocGenerics_0.36.0
## [49] limma_3.46.0
## [50] kableExtra_1.3.1
```

Table S1: Session Information and R Packages

```
##
## loaded via a namespace (and not attached):
##   [1] utf8_1.2.1                   R.utils_2.10.1
##   [3] tidyselect_1.1.0             RSQLite_2.2.3
##   [5] htmlwidgets_1.5.3            munsell_0.5.0
##   [7] codetools_0.2-16             preprocessCore_1.52.1
##   [9] statmod_1.4.35               withr_2.4.1
##  [11] colorspace_2.0-0             highr_0.8
##  [13] knitr_1.31                   rstudioapi_0.13
##  [15] labeling_0.4.2               GenomeInfoDbData_1.2.4
##  [17] farver_2.1.0                 bit64_4.0.5
##  [19] rhdf5_2.34.0                 vctrs_0.3.6
##  [21] generics_0.1.0               xfun_0.20
##  [23] biovizBase_1.38.0            R6_2.5.0
##  [25] illuminaio_0.32.0            AnnotationFilter_1.14.0
##  [27] bitops_1.0-6                 rhdf5filters_1.2.0
##  [29] cachem_1.0.3                 reshape_0.8.8
##  [31] DelayedArray_0.16.1          assertthat_0.2.1
##  [33] promises_1.1.1               scales_1.1.1
##  [35] bsseq_1.26.0                 nnet_7.3-14
##  [37] gtable_0.3.0                 ensembldb_2.14.0
##  [39] rlang_0.4.10                 splines_4.0.3
##  [41] rtracklayer_1.50.0           lazyeval_0.2.2
##  [43] DSS_2.38.0                   GEOquery_2.58.0
##  [45] dichromat_2.0-0              checkmate_2.0.0
##  [47] BiocManager_1.30.12          yaml_2.2.1
##  [49] GenomicFeatures_1.42.3       backports_1.2.1
##  [51] httpuv_1.5.5                 Hmisc_4.4-2
##  [53] tools_4.0.3                  nor1mix_1.3-0
##  [55] ellipsis_0.3.1               jquerylib_0.1.3
##  [57] siggenes_1.64.0              Rcpp_1.0.6
##  [59] plyr_1.8.6                   base64enc_0.1-3
##  [61] sparseMatrixStats_1.2.1      progress_1.2.2
##  [63] zlibbioc_1.36.0              BiasedUrn_1.07
```

Table S1: Session Information and R Packages

```
##  [65] purrr_0.3.4                  RCurl_1.98-1.2
##  [67] prettyunits_1.1.1            rpart_4.1-15
##  [69] openssl_1.4.3                RUVnormalizeData_1.10.0
##  [71] cluster_2.1.0                magrittr_2.0.1
##  [73] data.table_1.13.6            ProtGenerics_1.22.0
##  [75] mime_0.10                    hms_1.0.0
##  [77] evaluate_0.14                xtable_1.8-4
##  [79] XML_3.99-0.5                 jpeg_0.1-8.1
##  [81] readxl_1.3.1                 mclust_5.4.7
##  [83] compiler_4.0.3               biomaRt_2.46.3
##  [85] tibble_3.0.6                 KernSmooth_2.23-17
##  [87] crayon_1.4.1                 R.oo_1.24.0
##  [89] htmltools_0.5.1.1            later_1.1.0.1
##  [91] Formula_1.2-4                geneplotter_1.68.0
##  [93] tidyr_1.1.2                  DBI_1.1.1
##  [95] MASS_7.3-53                  rappdirs_0.3.3
##  [97] readr_1.4.0                  permute_0.9-5
##  [99] R.methodsS3_1.8.1            pkgconfig_2.0.3
## [101] GenomicAlignments_1.26.0     foreign_0.8-80
## [103] xml2_1.3.2                   annotate_1.68.0
## [105] bslib_0.2.4                  rngtools_1.5
## [107] multtest_2.46.0              beanplot_1.2
## [109] webshot_0.5.2                rvest_1.0.0
## [111] doRNG_1.8.2                  scrime_1.3.5
## [113] VariantAnnotation_1.36.0     digest_0.6.27
## [115] cellranger_1.1.0             rmarkdown_2.7
## [117] base64_2.0                   htmlTable_2.1.0
## [119] edgeR_3.32.1                 DelayedMatrixStats_1.12.3
## [121] curl_4.3                     shiny_1.6.0
## [123] Rsamtools_2.6.0              gtools_3.8.2
## [125] lifecycle_1.0.0              jsonlite_1.7.2
## [127] Rhdf5lib_1.12.1              askpass_1.1
## [129] BSgenome_1.58.0              fansi_0.4.2
## [131] pillar_1.5.1                 GO.db_3.12.1
```

Table S1: Session Information and R Packages

```
## [133] fastmap_1.1.0                    httr_1.4.2
## [135] survival_3.2-7                   interactiveDisplayBase_1.28.0
## [137] glue_1.4.2                       png_0.1-7
## [139] BiocVersion_3.12.0               bit_4.0.4
## [141] stringi_1.5.3                    sass_0.3.1
## [143] HDF5Array_1.18.1                 blob_1.2.1
## [145] DESeq2_1.30.0                    caTools_1.18.1
## [147] latticeExtra_0.6-29              memoise_2.0.0
## [149] dplyr_1.0.4
```
